# Comparison of the diagnostic efficacy between imaging features and iodine density values for predicting microvascular invasion in hepatocellular carcinoma

**DOI:** 10.3389/fonc.2024.1437347

**Published:** 2024-10-14

**Authors:** Jian Lv, Xin Li, Ronghua Mu, Wei Zheng, Peng Yang, Bingqin Huang, Fuzhen Liu, Xiaomin Liu, Zhixuan Song, Xiaoyan Qin, Xiqi Zhu

**Affiliations:** ^1^ Department of Radiology, Nanxishan Hospital of Guangxi Zhuang Autonomous Region, Guilin, China; ^2^ Graduate School, Guilin Medical University, Guilin, China; ^3^ Philips (China) Investment Co., Ltd., Guangzhou Branch, Guangzhou, China; ^4^ Department of Radiology, Affiliated Hospital of Youjiang Medical University for Nationalities, Baise, China; ^5^ Life Science and clinical Medicine Research Center, Affiliated Hospital of Youjiang Medical University for Nationalities, Baise, China

**Keywords:** hepatocellular carcinoma, microvascular invasion, spectral computer tomography, imaging features, iodine density

## Abstract

**Background:**

In recent years, studies have confirmed the predictive capability of spectral computer tomography (CT) in determining microvascular invasion (MVI) in patients with hepatocellular carcinoma (HCC). Discrepancies in the predicted MVI values between conventional CT imaging features and spectral CT parameters necessitate additional validation.

**Methods:**

In this retrospective study, 105 cases of small HCC were reviewed, and participants were divided into MVI-negative (n=53, Male:48 (90.57%); mean age, 59.40 ± 11.79 years) and MVI-positive (n=52, Male:50(96.15%); mean age, 58.74 ± 9.21 years) groups using pathological results. Imaging features and iodine density (ID) obtained from three-phase enhancement spectral CT scans were gathered from all participants. The study evaluated differences in imaging features and ID values of HCC between two groups, assessing their diagnostic accuracy in predicting MVI occurrence in HCC patients. Furthermore, the diagnostic efficacy of imaging features and ID in predicting MVI was compared.

**Results:**

Significant differences were noted in the presence of mosaic architecture, nodule-in-nodule architecture, and corona enhancement between the groups, all with p-values < 0.001. There were also notable disparities in IDs between the two groups across the arterial phase, portal phase, and delayed phases, all with p-values < 0.001. The imaging features of nodule-in-nodule architecture, corona enhancement, and nonsmooth tumor margin demonstrate significant diagnostic efficacy, with area under the curve (AUC) of 0.761, 0.742, and 0.752, respectively. In spectral CT analysis, the arterial, portal, and delayed phase IDs exhibit remarkable diagnostic accuracy in detecting MVI, with AUCs of 0.821, 0.832, and 0.802, respectively. Furthermore, the combined models of imaging features, ID, and imaging features with ID reveal substantial predictive capabilities, with AUCs of 0.846, 0.872, and 0.904, respectively. DeLong test results indicated no statistically significant differences between imaging features and IDs.

**Conclusions:**

Substantial differences were noted in imaging features and ID between the MVI-negative and MVI-positive groups in this study. The ID and imaging features exhibited a robust diagnostic precision in predicting MVI. Additionally, our results suggest that both imaging features and ID showed similar predictive efficacy for MVI.

## Introduction

The landscape of hepatocellular carcinoma (HCC) epidemiology is evolving with the surge in alcohol consumption, the escalating rates of obesity, and advancements in the treatment of hepatitis B virus and hepatitis C virus (1). Nevertheless, the implications of these shifts on the worldwide burden of HCC are not yet fully understood (1). HCC exhibits considerable heterogeneity and a grim prognosis, positioning it as the third most prevalent cause of cancer-related mortality globally (2). Surgical resection and liver transplantation remain the most effective treatments; nevertheless, recurrence stands out as a significant factor impacting the long-term survival of patients post-surgery (3).Tumor microvascular invasion (MVI) is a pivotal factor contributing to recurrence and poor prognosis post-HCC surgery, significantly affecting patient outcomes. For instance, in HCC patients with a tumor diameter greater than 2 cm and complicated by MVI, simply increasing the resection margin is insufficient to enhance overall survival rates (4). Despite efforts to widen the resection margin, long-term prognosis remains bleak, necessitating the implementation of additional comprehensive treatments to combat tumor recurrence and metastasis (4, 5). Therefore, the crucial assessment for MVI before cancer resection surgery profoundly influences the surgical approach, prognosis, and postoperative management. Consequently, the accurate evaluation of MVI in patients with primary small HCC has increasingly captured attention (6).

MVI is currently defined as a pathological condition distinguished by the presence of tumor cell nests along the walls of vascular structures, including endothelial cells within the portal and hepatic venous systems (7). To date, the conventional approach for preoperatively diagnosing HCC with MVI is through a pathological needle biopsy. However, needle biopsy presents several drawbacks, including invasiveness, a high risk of metastasis, a significant rate of false negatives, and challenges in acquiring adequate material, rendering it scarcely utilized in clinical settings (8). In recent years, numerous investigations have explored the association between laboratory markers, imaging features, and other parameters with MVI, yielding promising outcomes (9). Tumor imaging features, such as include non-smooth tumor margins, peritumoral enhancement and corona enhancement, have been extensively researched and utilized for predictive MVI due to their ability to reflect aspects of the tumor’s aggressive biological behavior (10). However, preoperative MVI diagnosis is challenging, and the imaging findings and definition are subjective and highly readerdependent. In recent years, studies have confirmed the predictive capability of spectral CT in determining MVI in patients with HCC (11–14). A study believed that conventional CT imaging features and spectral CT quantitative parameters exhibited almost identical predictive capabilities in anticipating MVI (11). Another study advocated that spectral CT offered more quantitative parameters compared to conventional CT, enhancing the differentiation between small HCC with and without MVI (12). Discrepancies in the predicted MVI values between conventional CT imaging features and spectral CT parameters necessitate additional validation.

This study aim to compare the diagnostic efficacy of imaging features and iodine density (ID) value, a parameter derived from spectral CT, for predicting MVI in HCC. In this study, 105 cases of small HCC were retrospectively reviewed, and subjects were categorized into MVI-negative and MVI-positive groups based on pathological findings. Imaging features and ID derived from three phase enhancement spectral CT scans were collected from all subjects. In this study, we assessed the discrepancies in imaging features and ID values of HCC between groups with and without MVI. We also explored the diagnostic accuracy of these imaging features and ID values in predicting the occurrence of MVI in HCC patients. Additionally, the diagnostic efficacy of imaging features and ID for predicting MVI were compared.

## Methods

**Figure 1:**
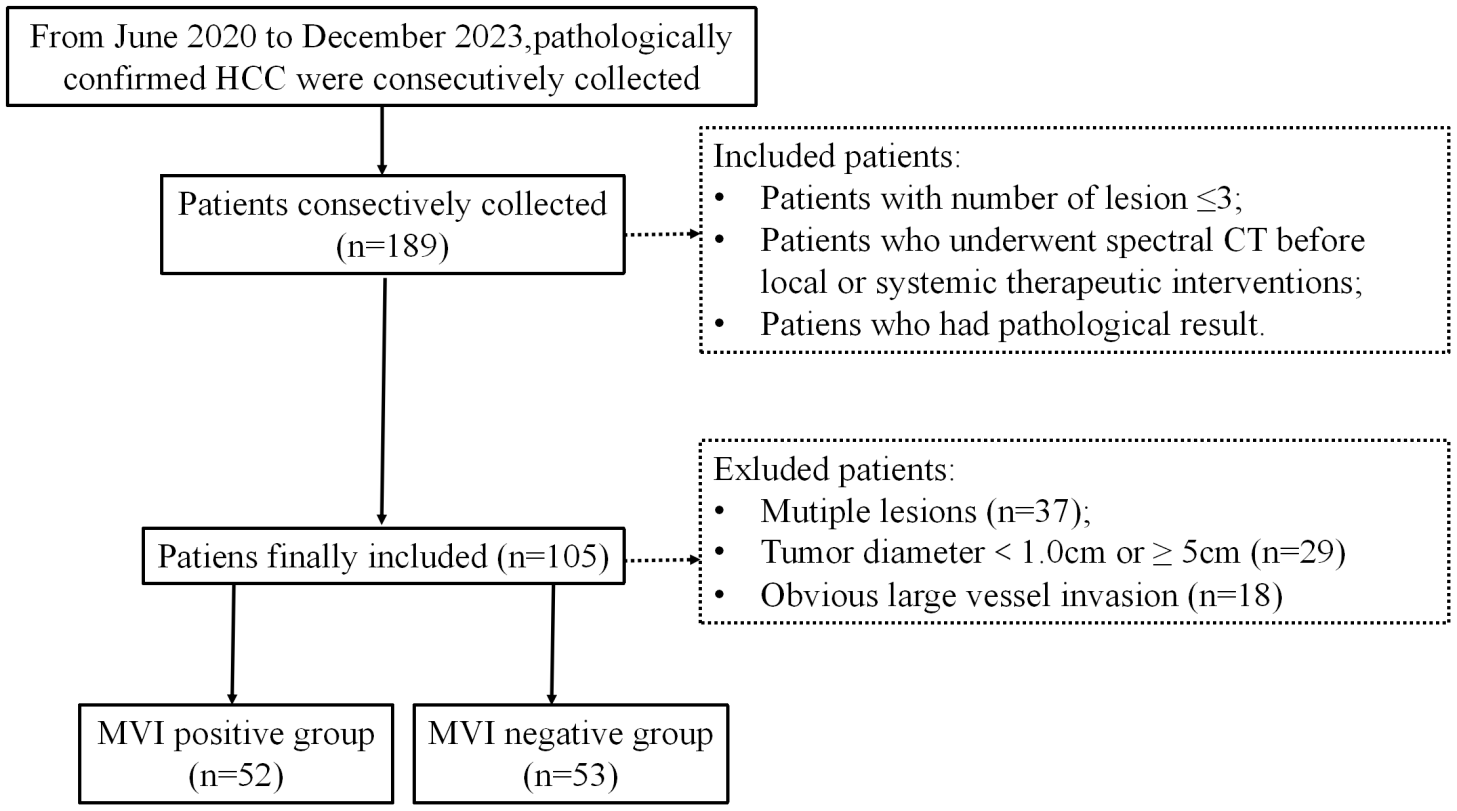
Flowchart shows inclusion and exclusion process of the subjects. HCC, hepatocellular cancer; MVI, microvascular invasion.

This is a retrospective study conducted at Nanxishan Hospital of Guangxi Zhuang Autonomous Region. Between June 2020 and July 2023, a total of 105 cases of HCC were collected, confirmed through pathological examination with surgical specimens in a consecutive manner. All patients had undergone spectral CT examination within 1 week before surgery. The inclusion and exclusion criteria for this study were detailed in **Figure 1**. The study was conducted in accordance with the Declaration of Helsinki (as revised in 2013). This study has been approved by the Ethics Committee of Nanxi Mountain Hospital, Guangxi Zhuang Autonomous Region [NO.2020 (KY-E-37)]. Due to its retrospective study, the requirement for informed consent is waived.

### Protocol of CT examination

CT scans were performed at a spectral CT scanner (IQon, Philips Healthcare, Amsterdam, The Netherlands). All scans included conventional CT, and contrast-enhanced scans (including arterial, portal and delayed phase). All patients drink about 250ml water before the inspection. The contrast agent (320mg/ml, 70 mL, iopromide; Beijing Beilu Pharmaceutical Co., Ltd., Beijing, China) was administered via an antecubital vein at an intended flow rate of 2.0~3.0ml/s. Scans were performed using the bolus chase method. The region of interest (ROI) was located at the same level of descending aorta. The arterial phase scan was performed after a 6S delay when the CT value exceeded the threshold of 180HU. A portal phase scan was performed after the arterial phase at the 33S. Delayed phase scanning was performed 101s after the arterial period. The same scanning protocol was repeated once during follow-up after TACE treatment in HCC patients. Protocols and parameters of the CT examination were shown as follows. Various phases of the CT liver examination were conducted using the following parameters. Common technical settings across all phases included a pitch of 1.234, rotation time of 0.27s, collimation size of 64×0.625, a field of view (FOV) of 345mm×345mm, a matrix size of 512×512, reconstruction slice and interval of 1mm, and employed the iDose4 level 4 reconstruction mode. Spectral parameters specific to Arterial, Portal and Delayed phases utilized Spectral-Based Imaging (SBI) spectral parameters in analysis.

### Imaging features analysis

Preoperative images were obtained from a picture archiving and communication system and independently reviewed by two faculty abdominal radiologists with over 10 (JL) and 5 years (RM) of experience. In cases of inter-reviewer discrepancies, a third observer (QX, with 15 years of experience) facilitated consensus. Though informed of the HCC diagnosis, all three readers remained blinded to clinical history and the pathological assessment of MVI. Following the principles of major and ancillary LI-RADS categorization, the identification of HCC high-risk patients involved assessing specific imaging features (15), including (a) lesion major diameter, (b) nonrim arterial phase hyperenhancement (nonrim APHE), (c) nonperipheral “washout,” (d) enhancing capsule, (e) mosaic architecture, (f) nodule-in-nodule architecture, (h) corona enhancement, and two non-LIRADS features: tumor nonsmooth tumor margin and intratumor necrosis (11, 16, 17). A detailed summary of CT imaging features is provided in **Table 1**.

**Table 1:** Comparison of demographic data and variables between two groups.

variables	MVI		t/χ2	*p-Value*
	Negative group, n(53)	Positive group, n(52)		
Clinical characteristics				
Male (n, %)	48(90.57)	50(96.15)	1.317	0.251
Age, years, mean ± SD	58.74 ± 9.21	59.40 ± 11.79	-0.324	0.747
imaging features				
Major diameter, cm, mean ± SD	5.94 ± 4.59	8.70 ± 4.22	-3.208	0.002**
Nonrim APHE (n, %)	50(94.34)	47(90.38)	0.583	0.445
Nonperipheral washout (n, %)	47(88.68)	49(94.23)	1.032	0.310
Enhancing capsule (n, %)	27(50.94)	36(69.23)	3.657	0.056
Mosaic architecture (n, %)	8(15.09)	23(44.23)	10.709	0.001**
Nodule-in-nodule architecture (n, %)	9(16.98)	36(69.23)	29.260	<0.001***
Corona enhancement (n, %)	11(20.75)	36(69.23)	24.946	<0.001***
Nonsmooth tumor margin (n, %)	10(18.87)	36(69.23)	27.045	<0.001***
Intratumor necrosis (n, %)	26(49.06)	44(84.62)	14.935	<0.001***
Conventional CT value, Hu, mean ± SD				
Arterial phase	25.44 ± 12.58	24.56 ± 12.87	0.357	0.722
Portal venous phase	38.98 ± 12.06	39.12 ± 11.69	-0.058	0.954
Delayed phase	30.75 ± 10.69	31.62 ± 9.83	-0.437	0.663
ID, mg/ml, mean ± SD				
ID-A	0.95 ± 0.30	1.49 ± 0.48	-6.945	<0.001***
ID-P	1.37 ± 0.34	1.98 ± 0.52	-7.121	<0.001***
ID-D	1.09 ± 0.20	1.55 ± 0.46	-6.611	<0.001***

Qualitative data are expressed as n (%). Chi-square test was used for comparisons among groups. Quantitative data are expressed as mean ± SD. Independent sample t-test was used for comparisons among groups. Statistical significance (P<0.05, **, p < 0.01. *** p< 0.001.)

MVI, microvascular invasion, APHE, arterial phase hyperenhancement; CT, computer tomography; ID-A, iodine density in hepatic arterial phase; ID-P, iodine density in portal-venous phase; ID-P, iodine density in delayed phase; Statistically significant results are marked in bold.

### ID analysis

**Figure 2:**
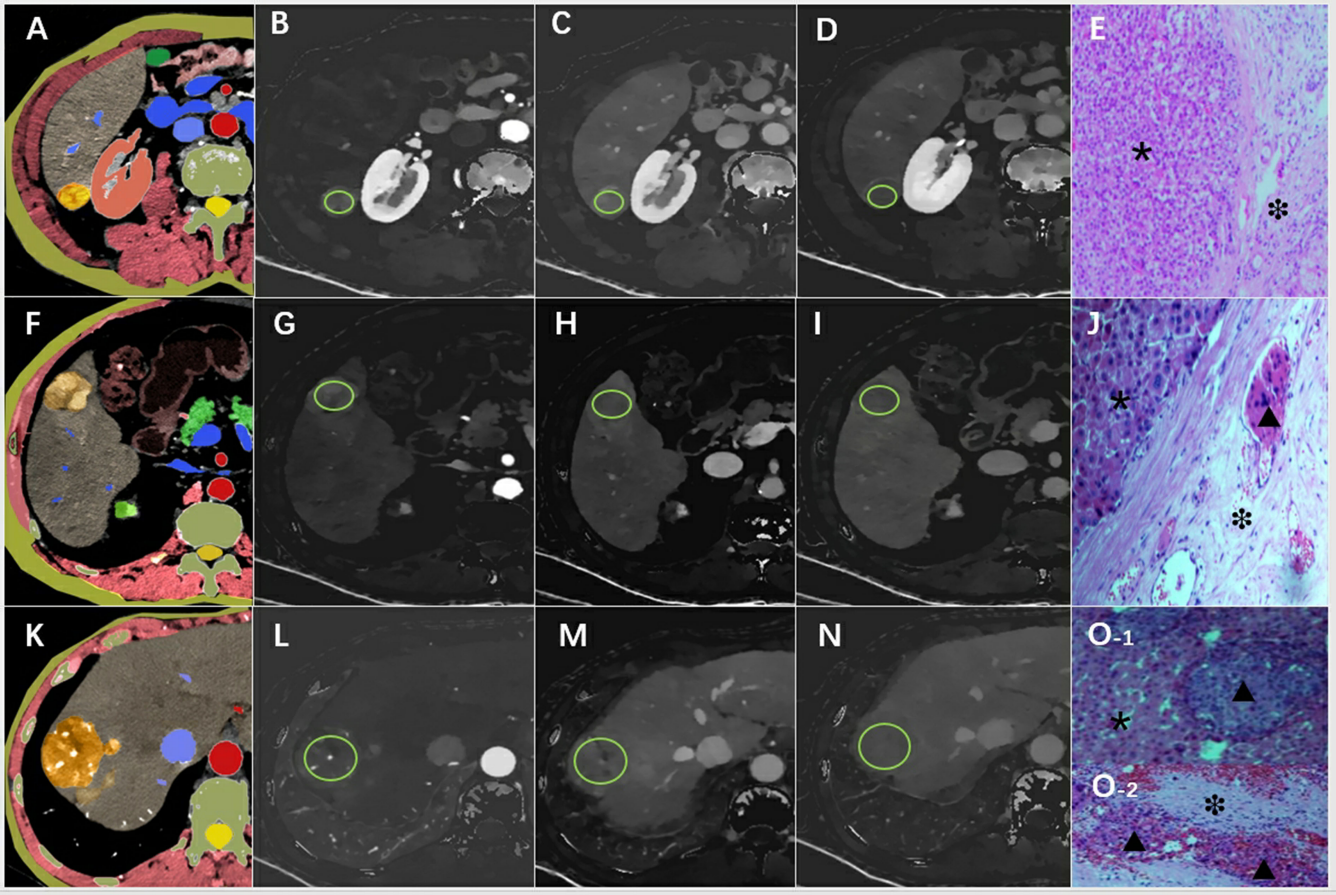
An example of the definition of the regions of interest. Examples of the definition of the regions of interest (ROIs) (light green circles) for quantitative analyses. **(A)** Schematic diagram of hepatocellular carcinoma with smooth tumor margin, **(B)** ID-A=1.02 mg/ml, **(C)** ID-P=1.23 mg/ml, **(D)** ID-D=1.05 mg/ml, **(E)** pathological pictures (×200), the tumor area (*) and MVI-negative in the normal area (❉) around the tumor were marked. **(F)** Schematic diagram of hepatocellular carcinoma with nodule-in-nodule architecture, **(G)** ID-A=1.87 mg/ml, **(H)** ID-P=1.58 mg/ml, **(I)** ID-D=1.32 mg/ml, **(J)** pathological pictures (×200) showed the tumor area (*) and MVI-positive(▴) in the normal area(❉) around the tumor. **(K)** Schematic diagram of hepatocellular carcinoma with corona enhancement, **(L)** ID-A=1.64 mg/ml, **(M)** ID-P=1.43 mg/ml, **(N)** ID-D=1.02 mg/ml, **(O)** pathological pictures (×200), O-1 shoued MVI-positive (▴) in the tumor (*), and also combined with MVI-positive (▴) in normal area (❉) in the O-2. ID-A, iodine density in hepatic arterial phase; ID-P, iodine density in portal-venous phase; ID-D, iodine density in delayed phase.

Two radiologists (JL and XQ, with over 10 and 15 years of experience in abdominal CT diagnosis, respectively), blinded to patients’ clinical data and pathological results, processed and measured all spectral CT images using the Philips Spectral Diagnostic Suite 9.0 workstation (Philips Healthcare). Three consecutive image slices, encompassing the maximum cross-section of the tumor and its upper and lower layers, were selected for region of interest (ROI) delineation and parameter measurement (**Figure 2**). The ROIs were drawn as large as possible to cover the entire cross-section of the tumor. Initially, the ROI was outlined on the arterial phase image of the spectral CT, and then it was transferred to the portal and delayed phase images of the spectral CT. ID values were automatically generated. Care was taken to place the ROI on solid tumor regions, avoiding areas with vessels, calcification, or cystic/necrotic changes.

### MVI examination

All surgical specimens were examined by one pathologist (L.Z., with 18 years of experience) who was blinded to the clinical and imaging data. A tissue sample containing the suspected tumor and surrounding tissue is acquired via biopsy or surgical resection and then fixed in formalin to maintain its structural integrity. Subsequently, the fixed tissue is dehydrated, embedded in paraffin, and sectioned thinly for microscopic examination. Following staining with hematoxylin and eosin (H&E) to visualize cellular details, all specimen sections are reviewed by a single experienced pathologist blinded to imaging and clinical data. Microscopic examination is conducted to detect any signs of microvascular invasion, characterized by tumor cells infiltrating small blood vessels or lymphatic channels near the tumor (**Figure 2**). The pathologist documents the presence, extent, and specifics of MVI in the pathology report and integrates these findings with other histopathological features to offer an accurate diagnosis and prognosis (18). Stringent quality control procedures, including potential review by multiple pathologists, are implemented to ensure the precision and reliability of the pathological assessments. The pathological assessment and diagnostic criteria for MVI in this study align with the Chinese Guidelines for the Diagnosis and Treatment of Hepatocellular Carcinoma (18).

### Statistical analysis

Statistical analyses were conducted using SPSS 26.0 (IBM Corp., Armonk, NY, USA). A bilateral p-value of less than 0.05 was considered indicative of statistical significance. The intraclass coefficient coefficient (ICC) test was utilized to assess the consistency of the ROI measurements made by both radiologists. Intraobserver agreements of imaging features were evaluated using Cohen κ-statistics. The strength of agreement via ICC and κ values < 0.4, 0.4–0.6, 0.6–0.8, and > 0.8 were categorized poor, moderate, good, and excellent agreement, respectively (13). The means of the variables measured by the two radiologists were utilized for statistical analysis. Normality and homogeneity of variance were tested using the Shapiro-Wilk test. Count data were presented as cases, and differences between groups were assessed using the chi-square test. Measurement data with a normal distribution were expressed as mean ± standard deviation and were compared between groups using an independent samples t-test, with *post-hoc* tests corrected using Bonferroni adjustment. Binary logistic regression analysis was employed to investigate the association between baseline spectral CT parameters and the presence of MVI. Initially, variables showing statistical associations were identified through binary logistic regression. Subsequently, variables with a p-value less than 0.05 were included in the multivariable logistic regression model to determine combined predictive parameters. Three prediction models were developed based on these combined parameters: Model I using imaging features, Model II based on spectral CT variables (including ID values on arterial, portal, and delayed phase images), and Model III incorporating both imaging features and spectral CT parameters. The diagnostic performance of spectral parameters and the three combined models was assessed using the receiver operating characteristic curve (ROC). Area under the curve (AUC) values, cutoff values, sensitivity, and specificity were calculated based on the optimal Youden index. Additionally, the DeLong test was employed to compare the AUC differences among the variables and models.

## Results

### Patient characteristics

Finally, a total of 105 patients with HCC were included in this study. Based on the presence or absence of MVI in the pathological results, all subjects were categorized into either the MVI-positive or MVI-negative group. The MVI-positive group comprised 53 subjects with a mean age of 59.40 ± 11.79 years and was predominantly male (50 individuals, 96.15%). In contrast, the MVI-negative group consisted of 52 subjects with a mean age of 58.74 ± 9.21 years and was also predominately male (48 individuals, 90.57%). There was no statistical difference in gender or age between the two groups. The baseline characteristics of patients are listed in **Table 1**.

### Reliability of measurements

The measurements obtained by both observers exhibited high consistency (P>0.05). The ICC values for the ID in the arterial, portal, and delayed phases were determined to be 0.803, 0.891, and 0.817, respectively. In imaging features, interobserver agreements were good to excellent, respectively (κ values range from 0.674 to 0.817).

### Comparison of variables between two groups

**Figure 3:**
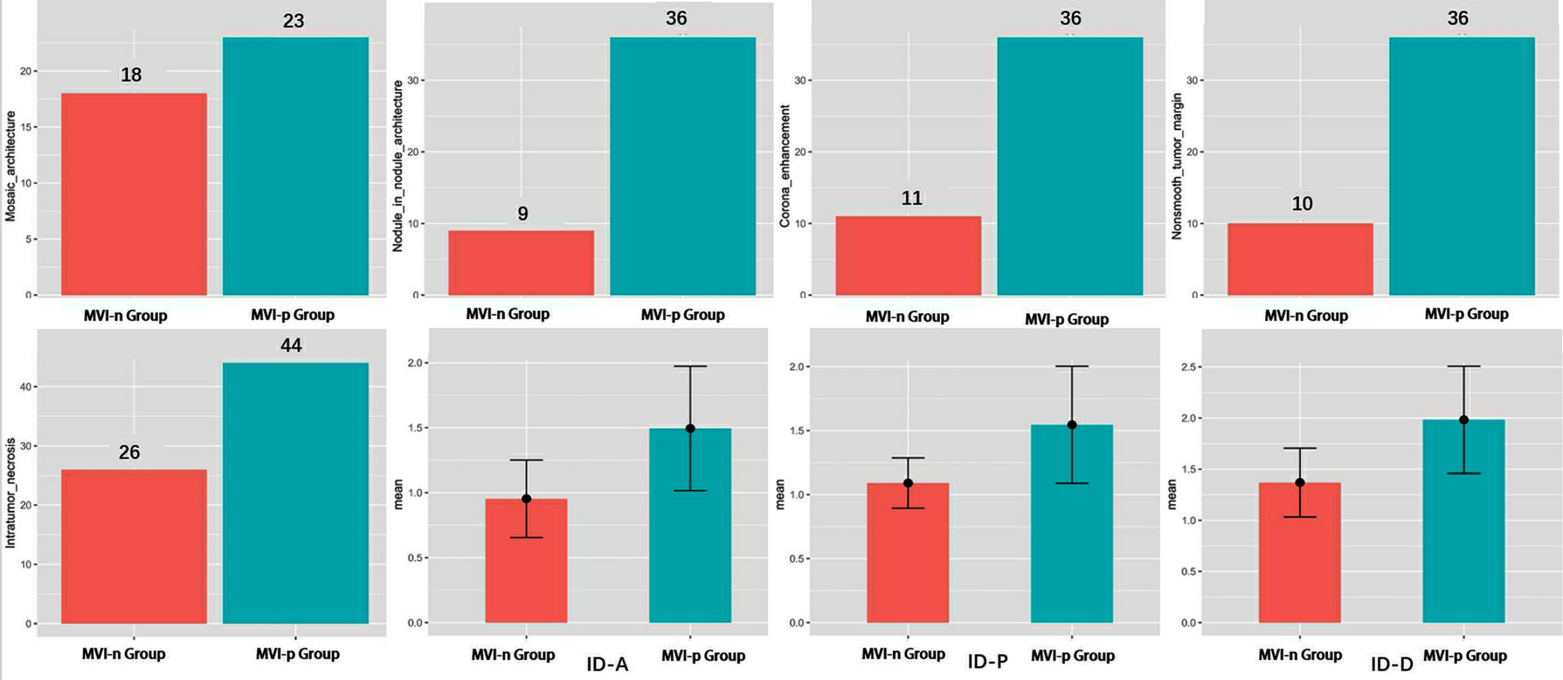
Analysis of differences in variables between two groups. MVI, microvascular invasion; ID-A, iodine density in hepatic arterial phase; ID-P, iodine density in portal-venous phase; ID-D, iodine density in delayed phase.

A detailed comparison of the imaging features between the two groups is provided in **Table 1** and **Figure 3**. The CT imaging features included the major diameter in centimeters with a mean ± SD of 5.94 ± 4.59 and 8.70 ± 4.22 for the MVI-negative and MVI-positive groups, respectively. The difference was statistically significant (p = 0.002). Other features such as nonrim APHE, nonperipheral washout, and enhancing capsule showed varying percentages without significant differences. Notable differences were observed in the presence of mosaic architecture, nodule-in-nodule architecture, corona enhancement, nonsmooth tumor margin, and intratumor necrosis between the groups, all with p-values < 0.001. However, conventional CT values in the arterial phase, portal venous phase, and delayed phase did not exhibit significant differences between the groups.

There was a significant difference in IDs between the two groups across the arterial phase, portal phase, and delayed phases (**Table 1** and **Figure 3**). The mean values of arterial phase ID in MVI-negative group (0.95 ± 0.30 mg/ml3) was significantly lower than that of MVI-positive group (1.49 ± 0.48 mg/ml^3^) (*p<*0.001). Similarly, the mean values of portal phase ID in MVI-negative group (1.37 ± 0.34 mg/ml^3^) was significantly lower than that of MVI-positive group (1.98 ± 0.52 mg/ml3) (*p<*0.001). Furthermore, the mean values of delayed phase ID in MVI-negative group (1.09 ± 0.20 mg/ml^3^) was significantly lower than that of MVI-positive group (1.55 ± 0.46 mg/ml3) (*p*=*<*0.001).

### The association between variables and MVI in HCC patients

**Figure 4:**
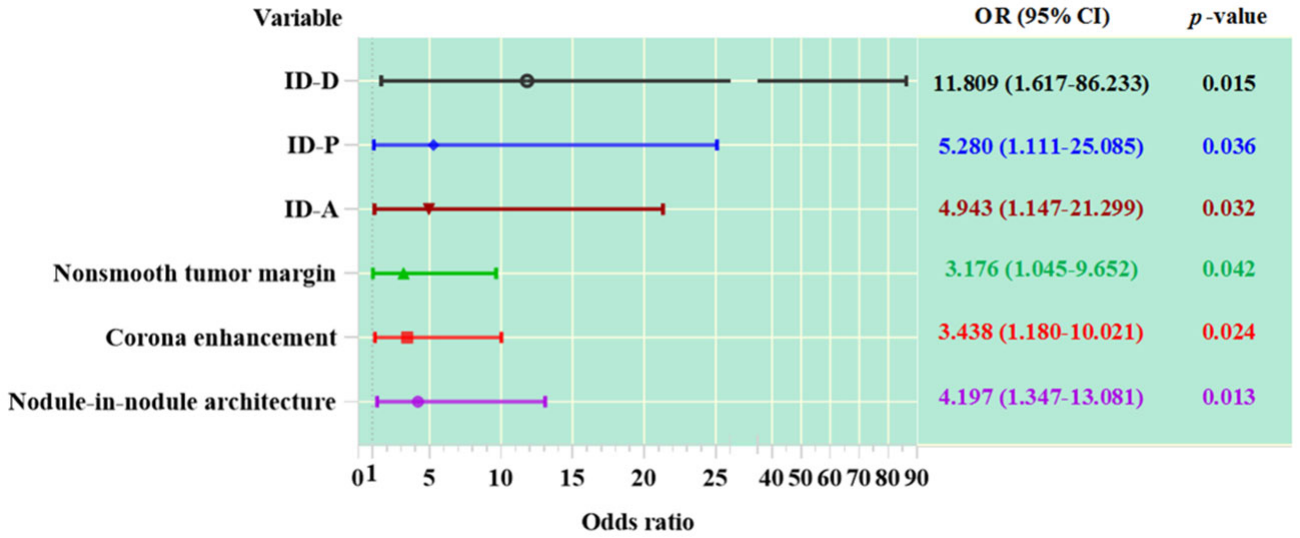
The association between variables and MVI in HCC patients. ID-A, iodine density in hepatic arterial phase; ID-P, iodine density in portal-venous phase; ID-D, iodine density in delayed phase.

Among the imaging features analyzed, nodule-in-nodule architecture, non-smooth tumor margin and corona enhancement demonstrated significant associations with the evaluation of MVI (see **Table 2** and **Figure 4**). The regression coefficient for nodule-in-nodule architecture was 1.434 (*p*=0.013), with an odds ratio (OR) of 4.197 (95% CI: 1.347-13.081) In contrast, the regression coefficient for corona enhancement was 1.235 (*p*=0.024), with an OR of 3.438 (95% CI: 1.180-10.021). Conversely, non-smooth tumor margin exhibited a regression coefficient of 1.156 (*p*=0.042), with an OR of 3.176 (95% CI: 1.045-9.652). Notably, other imaging features such as major diameter, mosaic architecture, and intratumor necrosis did not show a significant association with MVI.

**Table 2:** The association between variables and MVI.

variables	B	SE	*z* values	Wald χ2	*p-Value*	OR	95% CI
Imaging feature							
Major diameter, cm	-0.024	0.071	-0.344	0.118	0.731	0.976	0.849-1.121
Mosaic architecture	0.117	0.683	0.171	0.029	0.864	1.124	0.295-4.290
Nodule-in-nodule architecture	1.434	0.580	2.473	6.116	**0.013**	4.197	1.347-13.081
Corona enhancement	1.235	0.546	2.263	5.121	**0.024**	3.438	1.180-10.021
Nonsmooth tumor margin	1.156	0.567	2.038	4.152	**0.042**	3.176	1.045-9.652
Intratumor necrosis	1.174	0.639	1.836	3.372	0.066	3.234	0.924-11.317
ID							
ID-A	1.598	0.745	2.144	4.597	**0.032**	4.943	1.147-21.299
ID-P	1.664	0.795	2.093	4.380	**0.036**	5.280	1.111-25.085
ID-D	2.469	1.014	2.434	5.923	**0.015**	11.809	1.617-86.233

The association between variables and MVI of HCC patients were analyzed using binary logistic regression.

MVI, microvascular invasion; SE, standard error; OR, odds ratio; 95% CI, 95% confidence interval; ID-A, iodine density in hepatic arterial phase; ID-P, iodine density in portal-venous phase; ID-D, iodine density in delayed phase. Statistically significant results are marked in bold.

In patients with HCC, the values of ID in the arterial phase, portal phase, and delayed phase are significantly correlated with MVI (see **Table 2** and **Figure 4**). The regression coefficient for arterial phase ID was 1.598 (*p*=0.032), with an OR of 4.943 (95% CI: 1.147-21.299). Similarly, the regression coefficient for portal phase ID was 1.664 (*p*=0.036), with an OR of 5.280 (95% CI: 1.111-25.085). The delayed phase ID had a regression coefficient of 2.469 (*p*=0.015).

### The diagnostic efficacy of spectral CT parameters predicting MVI

Valuable insights from the analysis reveal that nodule-in-nodule architecture, corona enhancement, and nonsmooth tumor margin, as part of the general imaging features, exhibit significant diagnostic efficacy, boasting respective AUC of 0.761, 0.742, and 0.752. These imaging feature variables demonstrate notable sensitivity and specificity levels alongside significant *p*-values. Specifically, the diagnostic sensitivity for nodule-in-nodule architecture, corona enhancement, and nonsmooth tumor margin was 0.692, 0.692, and 0.692, respectively, while their diagnostic specificity values were 0.830, 0.792, and 0.811 correspondingly.

**Figure 5:**
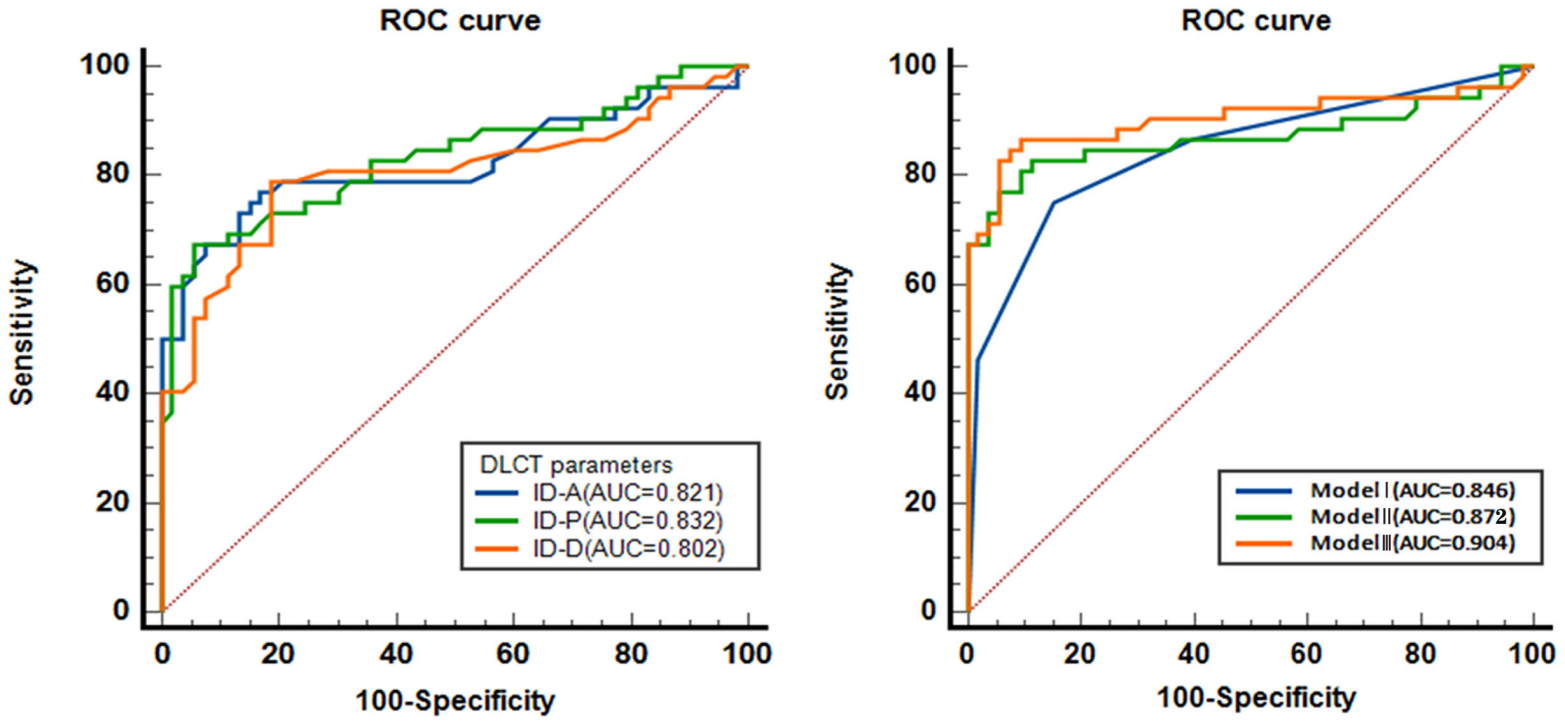
The diagnostic efficacy of spectral CT parameters predicting MVI. ROC, receiver operating characteristic curve. AUC, area under the curve; ID-A, iodine density in hepatic arterial phase; ID-P, iodine density in portal-venous phase; ID-D, iodine density in delayed phase. Model I, combination of the variables of General CT characteristics; Model II, combination of the variables of ID; Model III, combination of Model I and Model II.

In spectral CT analysis, the identifiers for arterial, portal, and delayed phase ID demonstrate significant diagnostic accuracy in detecting MVI. The arterial phase ID, at a cut-off value of 0.599 mg/ml^3^, yielded an AUC of 0.821 (95% CI: 0.734-0.907) with sensitivity and specificity values of 76.9% and 83.0%, respectively. Similarly, the portal phase ID, at a cut-off value of 0.616 mg/ml^3^, achieved an AUC of 0.832 (95% CI: 0.751-0.913) with sensitivity and specificity rates of 67.3% and 94.3%. Moreover, the delayed phase imaging contrast, with a cut-off value of 0.600 mg/ml^3^, demonstrated an AUC of 0.802 (95% CI: 0.713-0.891) coupled with sensitivity and specificity values of 78.8% and 81.1% respectively (**Table 3** and **Figure 5**).

**Table 3:** The diagnostic efficacy of spectral CT parameters for predicting MVI.

Variables/models	AUC	Cut-off values	Sensitivity	Specificity	*P- values*	95% CI
Imaging features						
Nodule-in-nodule architecture	0.761	/	0.692	0.830	<0.001***	0.667 ~ 0.856
Corona enhancement	0.742	/	0.692	0.792	<0.001***	0.645 ~ 0.839
Nonsmooth tumor margin	0.752	/	0.692	0.811	<0.001***	0.656 ~ 0.848
ID						
ID-A	0.821	0.599	0.769	0.830	<0.001***	0.734 ~ 0.907
ID-P	0.832	0.616	0.673	0.943	<0.001***	0.751 ~ 0.913
ID-D	0.802	0.600	0.788	0.811	<0.001***	0.713 ~ 0.891
Combined models						
Model I	0.846	/	0.750	0.849	<0.001***	0.769 ~ 0.923
Model II	0.872	0.714	0.827	0.887	<0.001***	0.795 ~ 0.949
Model III	0.904	/	0.865	0.925	<0.001***	0.836 ~ 0.973

The diagnostic efficacy of spectral CT parameters for predicting MVI were analyzed using receiver operating characteristic curve, ***, p<0.001.

AUC, area under the curve; 95% CI, 95% confidence interval; ID-A, iodine density in hepatic arterial phase; ID-P, iodine density in portal-venous phase; ID-D, iodine density in delayed phase. Model I, combination of the variables of General CT characteristics; Model II, combination of the variables of ID; Model III, combination of Model I and Model II.

Moreover, the combined models, including Model I, Model II, and Model III, show excellent AUC values, strong sensitivity and specificity, and significant predictive capabilities. The AUC of combined model I was 0.846 (95% CI: 0.769 ~ 0.923), with a sensitivity and specificity of 75.0% and 84.9%, respectively. The AUC of combined model II was 0.872 (95% CI: 0.795 ~ 0.949), with a sensitivity and specificity of 82.7% and 88.7%, respectively. The AUC of combined model III was 0.904 (95% CI: 0.836 ~ 0.973), with a sensitivity and specificity of 86.5% and 92.5%, respectively (**Table 3** and **Figure 5**).

### DeLong test between AUCs

A comparison of the DeLong test among the AUCs was outlined in **Table 4**. The DeLong test results revealed statistically significant differences between nodule-in-nodule architecture and Model I (*p*=0.0061), corona enhancement and Model I (*p*=0.0019), nonsmooth tumor margin and Model I (*p*=0.0029), as well as between Model III and Model I (*p*=0.0459).

**Table 4:** The comparison of diagnostic efficacy between different variables and combined models.

AUC	D-value	SE	95% CI	*z* values	*p* values
Imaging features					
Nodule-in-nodule architecture vs. Corona enhancement	0.019	0.051	-0.081 ~ 0.118	0.371	0.710
Nodule-in-nodule architecture vs. Nonsmooth tumor margin	0.009	0.048	-0.085 ~ 0.104	0.196	0.845
Corona enhancement vs. Nonsmooth tumor margin	0.009	0.052	-0.092 ~ 0.111	0.182	0.855
Nodule-in-nodule architecture vs. ModelI	0.084	0.031	0.024 ~ 0.145	2.742	**0.006**
Corona enhancement vs. ModelI	0.103	0.033	0.038 ~ 0.168	3.105	**0.002**
Nonsmooth tumor margin vs. ModelI	0.094	0.032	0.032 ~0.155	2.979	**0.003**
ID					
ID-A vs.ID-P	0.011	0.042	-0.070 ~ 0.093	0.275	0.783
ID-A vs.ID-D	0.019	0.051	-0.080 ~ 0.117	0.366	0.714
ID-A vs.ModelII	0.050	0.030	-0.009 ~0.109	1.676	0.094
ID-P vs.ID-D	0.030	0.044	-0.057 ~ 0.117	0.675	0.500
ID-P vs.ModelII	0.039	0.023	-0.005 ~ 0.083	1.735	0.083
ID-D vs.ModelII	0.069	0.035	0.000 ~ 0.138	1.947	0.052
Combined models					
ModelIvs.ModelII	0.025	0.043	-0.058 ~ 0.114	0.595	0.552
ModelIvs.ModelIII	0.058	0.029	0.001 ~ 0.114	1.996	**0.046**
ModelIIvs.ModelIII	0.032	0.022	-0.012 ~0.076	1.437	0.151

The difference of diagnostic efficacy between different Variables and combined models were analyzed using Delong-test. The bold values indicate p < 0.05.

AUC, area under the curve; D-value, The difference in AUC values; SE, standard error; 95% CI, 95% confidence interval; SD, standard deviation; cm, centimeter; ID-A, iodine density in hepatic arterial phase; ID-P, iodine density in portal-venous phase; ID-D, iodine density in delayed phase. Model I, combination of the variables of General CT characteristics; Model II, combination of the variables of ID; Model III, combination of Model I and Model II.

## Discussion

In this study, we found that both ID values and imaging features of HCC exhibited a robust diagnostic accuracy in predicting MVI. Interestingly, no significant disparity was observed in the diagnostic efficacy of ID values and imaging features for predicting MVI.

### Imaging features in MVI prediction

In the present study, the incidence of imaging features such as nodule-in-nodule architecture, corona enhancement, and nonsmooth tumor margin was lower in the MVI-negative group than the MVI-positive group. These imaging characteristics mirror, to some extent, the molecular biological characteristics and aggressiveness of liver cancer. Corona enhancement, indicative of the tumor’s elevated blood flow perfusion, is commonly associated with increased arterial input and venous output surrounding the tumor (19). The existence of nodule-in-nodule structures offers compelling evidence of the multistep progression in hepatocarcinogenesis, and indicating a transformation in HCC biology (20). The imaging feature of nonsmooth tumor margin can be regarded as indicative of HCC’s aggressive biological tendencies to invade the tumor capsule and protrude into the non-tumoral parenchyma (21). These imaging feature probably relates to the known hypothesis of tumor aggressive characteristics that aid in identifying MVI. Previous studies have established a positive correlation between corona enhancement and MVI. Lee and colleagues demonstrated that corona enhancement serves as an imaging biomarker for predicting the occurrence of MVI (22). Yang et al. reported that, as the preoperative biomarkers for predicting MVI, corona enhancement demonstrates the highest accuracy, surpassing both internal arteries and tumor size (23. A non-smooth tumor margin is a promising indicator of preoperative MVI assessment. Li et al. reported the non-smooth tumor margin and tumor size were significantly associated with MVI (24). Jiang et al. discovered that a non-smooth tumor margin exhibits a stronger association with MVI when compared to other imaging features, including internal artery, hepatobiliary phase peritumoral hypointensity, and tumor multifocality (25). In a meta-analysis of non-smooth tumor margin predicting MVI, the pooled AUC, sensitivity and specificity were 0.79, 35% and 79%, respectively (21). A nodule-in-nodule appearance in early-stage HCC could be interpreted as a morphologic marker of dedifferentiation of early HCC (26). However, this imaging feature is controversial when link it to MVI. Sheng et al. found that the imaging feature of nodule-in-nodule architecture showed a significant association with MVI, with a magnitude of association comparable to that of the internal artery (27). The study by Wang et al. found that there is no significant correlation between the imaging feature of nodule-in-nodule and MVI (28). Moreover, another study reported that absence of nodule-in-nodule architecture were independent predictors of MVI, and the association with MVI was significantly higher than tumor size (29). Although the diagnostic efficacy of imaging features in predicting MVI is well-established, concerns persist regarding the predictability and repeatability of these features. Moreover, there is still controversy surrounding their positive predictive values (30). Several factors may contribute to the inconsistency. Firstly, the efficacy of these imaging features in predicting MVI can vary across different studies due to factors such as sample sizes and patient demographics (31). Secondly, precise identification and interpretation of imaging features are essential for ensuring repeatability since image interpretation hinges on both the quality of the device and the proficiency of the operator. For instance, corona enhancement may be mistaken for a peritumoral capsule due to its appearance, and likewise, mosaic architecture could be misconstrued as necrosis (32). A sensitivity of only 54% is observed in imaging diagnosis of non-smooth tumor margins compared to pathological diagnosis (33). In future study, a third radiologist was consulted to obtain consensus evaluation may reduce the probability of imaging feature misinterpretation.

### Spectral CT metrics in MVI prediction

In this study, we found the disparity of ID values between groups and validated that ID values have a strong diagnostic accuracy in predicting MVI. Spectral CT parameters have been validated as a surrogate method for assessing perfusion abnormalities in organs, facilitating the evaluation of tumor grading, pathological classification, and invasiveness by detecting changes in tumor perfusion (34–37). ID plays a pivotal role in predicting the aggressiveness and tumor characteristics of HCC. This metric provides valuable information about vascular permeability, tumor perfusion, and tissue iodine uptake, offering insights into the underlying pathophysiological mechanisms driving tumor behavior. Lewin et al. discovered that the spectral CT parameters model successfully predicted intratumoral and peritumoral MVI in HCC (14). Mulé et al. reported that in advanced HCC lesions, late-arterial ID is strongly related to both blood flow and blood volume, while portal ID mainly reflects blood volume, offering ID the ability to evaluate both morphological and perfusion changes (38). Zhu et al. employed spectral CT parameters to predict MVI in solitary AFP-negative HCC measuring ≤ 5 cm, achieving an AUC of 0.755 for arterial ID and 0.683 for delayed ID (11). In another study, Yang et al. employed spectral CT for evaluating MVI in small HCC ≤ 3 cm, reporting an AUC of 0.853, a sensitivity of 87.50%, and a specificity of 79.60% (12). Kim et al. evaluated the diagnostic performance of normalized intratumoral and peritumoral IDs in predicting MVI. Their findings revealed that the ID in outer layer 1 demonstrated the highest AUC of 0.747 (13). Our findings are in line with the above conclusions, demonstrating the consistent stability and repeatability of spectral CT parameters in predicting MVI.

### Comparison of imaging features and spectral CT parameters in predicting MVI

Another result of this study revealed no significant difference in the diagnostic efficacy between ID values and imaging features for predicting MVI in HCC. Moreover, the incorporation of both protocols did not improve the predictive accuracy of spectral CT. Zhu et al. observed that conventional CT imaging features and spectral CT quantitative parameters demonstrated nearly identical predictive abilities in predicting MVI, with AUCs of 0.848 and 0.849, respectively. Yet, the combination of imaging features and spectral CT parameters notably enhanced the diagnostic efficacy (11). In a study by Yang et al., spectral CT was found to offer more quantitative parameters compared to conventional CT, enhancing the differentiation between small HCC with and without MVI (12). Imaging feature has also been confirmed to improve the diagnostic efficacy of imaging omics in predicting MVI (23). The results of the aforementioned studies do not align with our findings. Further validation is needed to evaluate the supplementary predictive benefit of spectral CT parameters over conventional CT imaging features. In addition to its recognized superior predictive capability for MVI, spectral CT is acknowledged to provide further advantages in MVI prognostication. These benefits encompass heightened lesion detection, enhanced lesion characterization, improved imaging features interpretation, inherently robust imaging protocols for pathological explication, and reduced radiation exposure (39).

### Limitation

This study had several potential limitations. Firstly, this was a single center, single scanner, and single vendor retrospective study, and the sample size of this study is insufficient, The results of this study need to be verified by more diversity of this study. Secondly, this study is a retrospective study, and the study efficacy may receive the effect of case selection. Thirdly, In this study, we only used ID to predict MVI. The first reason is that spectral parameters are derived by the same decomposition model, other parameters does not provide additional lesion information rather than the ID (34). The second reason is that the risk of multicollinearity may increase using multiple spectral parameters derived from the same set of images in the same statistical model. Finally, in addition to imaging features and spectral CT, various protocols such as radiomics models, quantitative parameters of magnetic resonance imaging, and molecular imaging have shown promising predictive abilities for MVI (40–42). This study did not include a comparison with these models. Conducting further comparisons among different imaging schemes to predict MVI will be beneficial for clinicians in making informed decisions.

## Conclusion

Substantial differences were noted in imaging features and ID between the MVI-negative and MVI-positive groups in this study. The ID and imaging features exhibited a robust diagnostic precision in predicting MVI. Additionally, our results suggest that both imaging features and ID showed similar predictive efficacy for MVI. Additional validation is essential to ascertain the differences in predictive accuracy between imaging characteristics and spectral CT for MVI.

## Data Availability

The raw data supporting the conclusions of this article will be made available by the authors, without undue reservation.
